# Application of holographic imaging combined with real-time ultrasound-guided robot-assisted partial nephrectomy in the treatment of completely endophytic renal tumours: a retrospective cohort study comparing with pure laparoscopic surgery

**DOI:** 10.3389/fonc.2025.1671896

**Published:** 2025-11-24

**Authors:** Tongbin Gao, Yuan Gao, Dawei Wang, Yongchun Yu, Zhentao Zhang, Feihu Tang

**Affiliations:** 1 Weifang People’s Hospital, Urology Medical Center, Weifang, China; 2 Affiliated Hospital of Shandong Second Medical University, School of Clinical Medicine, Shandong Second Medical University, Weifang, China

**Keywords:** completely endophytic renal tumour, da Vinci robotic system, holographic imaging technology, intraoperative ultrasound, minimally invasive surgery

## Abstract

**Background:**

To compare the perioperative outcomes and renal function preservation between holographic imaging combined with real-time ultrasound-guided robotic-assisted partial nephrectomy (RAPN) using the Da Vinci system and conventional laparoscopic partial nephrectomy (LPN) for completely endophytic renal tumours, and to explore the clinical advantages of novel imaging technologies.

**Methods:**

A single-centre retrospective cohort study was conducted, including 61 patients with completely endophytic renal tumours treated at Weifang People’s Hospital from January 2022 to January 2025. All patients underwent holographic imaging and intraoperative ultrasound. They were divided into the RAPN group (n=31) and the LPN group (n=30), with balanced baseline characteristics (all *P>*0.05), Crucially, all patients in both groups underwent preoperative holographic imaging and intraoperative ultrasound. Primary outcomes included warm ischemia time (WIT), intraoperative blood loss, operative time, and postoperative estimated glomerular filtration rate (eGFR) changes. Secondary outcomes comprised positive surgical margin rates, complications, and oncologic outcomes.

**Results:**

Perioperative outcomes: WIT was reduced by 20% in the RAPN group (20 [IQR 20-25] *vs*. 25 [20-30] min, *P* = 0.037). Intraoperative blood loss distribution differed significantly (50 [20-50] *vs*. 50 [50-50] ml, *P* = 0.028), with 25% of RAPN cases achieving blood loss ≤20 ml (minimum in LPN: 50 ml). No statistical differences were observed in operative time (145 [126-193] *vs*. 133 [115-163] min) or hospital stay (7 [6-7] *vs*. 7 [5-7] days, both *P>*0.05). Complication rates were similar (9.7% *vs*. 6.7%, *P=*1.000). Renal function preservation: Postoperative eGFR on day 1 (84.67 ± 22.25 *vs*. 87.26 ± 19.92 ml/min, *P=*0.634) and at 3 months (92.17 ± 30.42 *vs*. 95.21 ± 22.91 ml/min, *P=*0.738) showed no significant differences. Oncologic safety: Both groups achieved 100% negative surgical margins, with a comparable distribution of malignant pathological subtypes and WHO/ISUP grades. No recurrence was detected during follow-up.

**Conclusion:**

The RAPN platform, when integrated with holographic imaging and real-time ultrasound guidance, demonstrates significant advantages in treating completely endophytic renal tumour. This integrated robotic approach offers a precise and minimally invasive solution for complex cases. Study limitations include its retrospective design, and a phase III RCT (N≥200) is recommended to validate long-term outcomes.

## 1 Introduction

Partial nephrectomy (PN) is the gold standard treatment for T1-stage renal tumours (1). The advancement of surgical techniques continues to push the boundaries of managing tumours with complex anatomical configurations. In recent years, the clinical management of completely endophytic tumours (those entirely surrounded by renal parenchyma) has emerged as a significant challenge in the field of urology. Innovations such as the Da Vinci robotic system, intraoperative ultrasound, and holographic imaging have provided novel solutions to this challenge. However, there remains a lack of high-level evidence comparing the efficacy of RAPN guided by holographic imaging and real-time ultrasound versus conventional LPN in treating completely endophytic tumours. This single-centre retrospective cohort study aims to: (1) quantitatively evaluate the differences in perioperative outcomes (e.g., WIT and blood loss) between the two techniques; (2) elucidate the potential advantages of new technologies in preserving renal function; and (3) establish clinical guidelines for the application of advanced imaging fusion technologies.

## 2 Subjects and methods

### 2.1 Study design and ethics

This retrospective study was approved by the Medical Research Ethics Committee of Weifang People’s Hospital (Approval No. KYLL20250721-7). The studies were conducted in accordance with the local legislation and institutional requirements. The participants provided their written informed consent to participate in this study.

### 2.2 Study subjects

From January 2022 to January 2025, a total of 65 patients with completely endophytic renal tumours were initially identified as potentially eligible for the study. After applying the inclusion and exclusion criteria, 63 patients were deemed suitable for analysis. Following 1:1 propensity score matching (PSM), 61 patients were ultimately included in the study cohort (RAPN group: n=31; LPN group: n=30).

Inclusion Criteria: Patients preoperatively diagnosed with completely endophytic renal tumours via contrast-enhanced abdominal CT/MRI (meeting all the following criteria):(1) tumour 100% embedded within renal parenchyma (“E” = 3 points in R.E.N.A.L. nephrometry score);(2) Clinical stage cT1 (maximum diameter ≤7 cm);(3) Treated with either RAPN (using the fourth-generation Da Vinci Xi robotic system) or LPN;(4) Intraoperative application of holographic imaging (provided by Ziwei Dixing Digital Medical Technology Co., Ltd.) and intraoperative ultrasound (SonoScape, Shenzhen Mindray Bio-Medical Electronics Co., Ltd.).

Exclusion Criteria:

(1) Imaging suggesting metastasis (M1) or regional lymph node metastasis (N1) (2); Loss to follow-up or incomplete postoperative data.

Supporting demographic context: According to the Seventh National Population Census of Weifang City (2020), the permanent population is 9,386,677. Based on the national crude incidence rate of kidney tumours in China (5.48 per 100,000) (2), it is estimated that approximately 510 new cases are diagnosed annually in Weifang City. Weifang People’s Hospital, as a regional tertiary referral centre, performs approximately 280 kidney tumour surgeries annually. Based on the reported incidence of completely endophytic renal tumours (approximately 10% of all renal tumors (3)), the expected number of such cases is about 28 per year. Therefore, the recruitment of 61 patients over 3 years is consistent with the epidemiological profile and clinical volume of our institution. A detailed patient screening and enrolment flowchart is provided as **Supplementary Figure S1** (**Supporting Information**).

### 2.3 Grouping and baseline data

To minimize selection bias and control for confounding variables, patients were divided into RAPN (n=31) and LPN (n=30) groups after 1:1 PSM. The propensity score was estimated using a logistic regression model with the surgical approach as the dependent variable. Matching was performed using the nearest-neighbour algorithm with a caliper width of 0.2 standard deviations of the logit of the propensity score. Matching covariates included age, gender, body mass index (BMI), history of hypertension, tumour size, R.E.N.A.L. nephrometry score, and preoperative eGFR.

This PSM process resulted in the exclusion of 2 patients from the LPN group in the original cohort (n=63) due to a lack of suitable matches within the predefined caliper width. The detailed baseline characteristics and specific reasons for exclusion for these two patients are provided in **Supplementary Table S1**. Importantly, a comparative analysis demonstrated that these excluded patients showed no significant differences from the final included cohort in all baseline characteristics (all P > 0.05, **Supplementary Table S2**), indicating that the exclusion process did not introduce substantial selection bias. After matching, standardized mean differences (SMD) for all covariates were reduced to below 0.1 (**Table 1**), indicating excellent balance between the two groups. Any residual minor imbalances in tumour size were addressed via multivariate adjustment in sensitivity analyses.

**Table 1:** Comparison of baseline characteristics between RAPN and LPN groups before and after propensity score matching.

Variable	Before PSM			After PSM			
	RAPN group (n=31)	LPN group (n=32)	SMD	RAPN group (n=31)	LPN group (n=30)	SMD	P-value
Age (years)	54.8 ± 14.0	57.2 ± 13.5	0.175	54.8 ± 14.0	56.5 ± 12.3	0.072	0.612
Male, n (%)	20 (64.5%)	22 (68.8%)	0.091	20 (64.5%)	20 (66.7%)	0.045	0.865
BMI (kg/m²)	25.1 ± 3.8	26.8 ± 4.9	0.382	25.1 ± 3.8	25.3 ± 4.2	0.051	0.847
Hypertension, n (%)	14 (45.2%)	13 (40.6%)	0.092	14 (45.2%)	12 (40.0%)	0.104	0.687
Tumor size (cm)	3.0 ± 0.8	2.6 ± 1.0	0.452	3.0 ± 0.8	2.5 ± 1.0	0.085	0.584
R.E.N.A.L. score	9 (8, 10)	8 (8, 9)	-	9 (8, 10)	8.5 (8, 10)	-	0.703
Preoperative eGFR (ml/min/1.73m²)	106.4 ± 16.5	103.8 ± 19.1	0.146	106.4 ± 16.5	105.0 ± 18.6	0.043	0.752

(SMD values <0.1 indicate excellent balance between groups after matching. The residual imbalance in tumor size (SMD = 0.085) was addressed in sensitivity analyses.).

All RAPN and LPN procedures were performed by the same experienced chief surgeon and their team to minimize variability in surgical skill. Patient allocation to RAPN or LPN was based on equipment availability and preoperative anatomical assessment (e.g., tumour proximity to renal vessels), as both techniques were considered standard options for eligible patients, not surgeon or patient preference.

### 2.4 Sample size justification

The sample size was estimated based on the primary outcome (WIT). Prior studies reported a mean WIT of 25 ± 10 minutes for LPN in endophytic tumours (4, 5). To detect a clinically relevant 5-minute reduction in WIT (20% improvement) with 80% power (α=0.05, two-tailed t-test), a minimum of 30 patients per group was required (effect size d=0.5, SD = 10 min). For secondary outcomes (e.g., complication rates), this sample provided 70% power to detect a 15% absolute difference (baseline rate 10% (6, 7)). Despite the rarity of completely endophytic tumours, our cohort (N = 61) aligns with similar comparative studies (4, 5).

### 2.5 Preoperative evaluation

**Figure 1:**
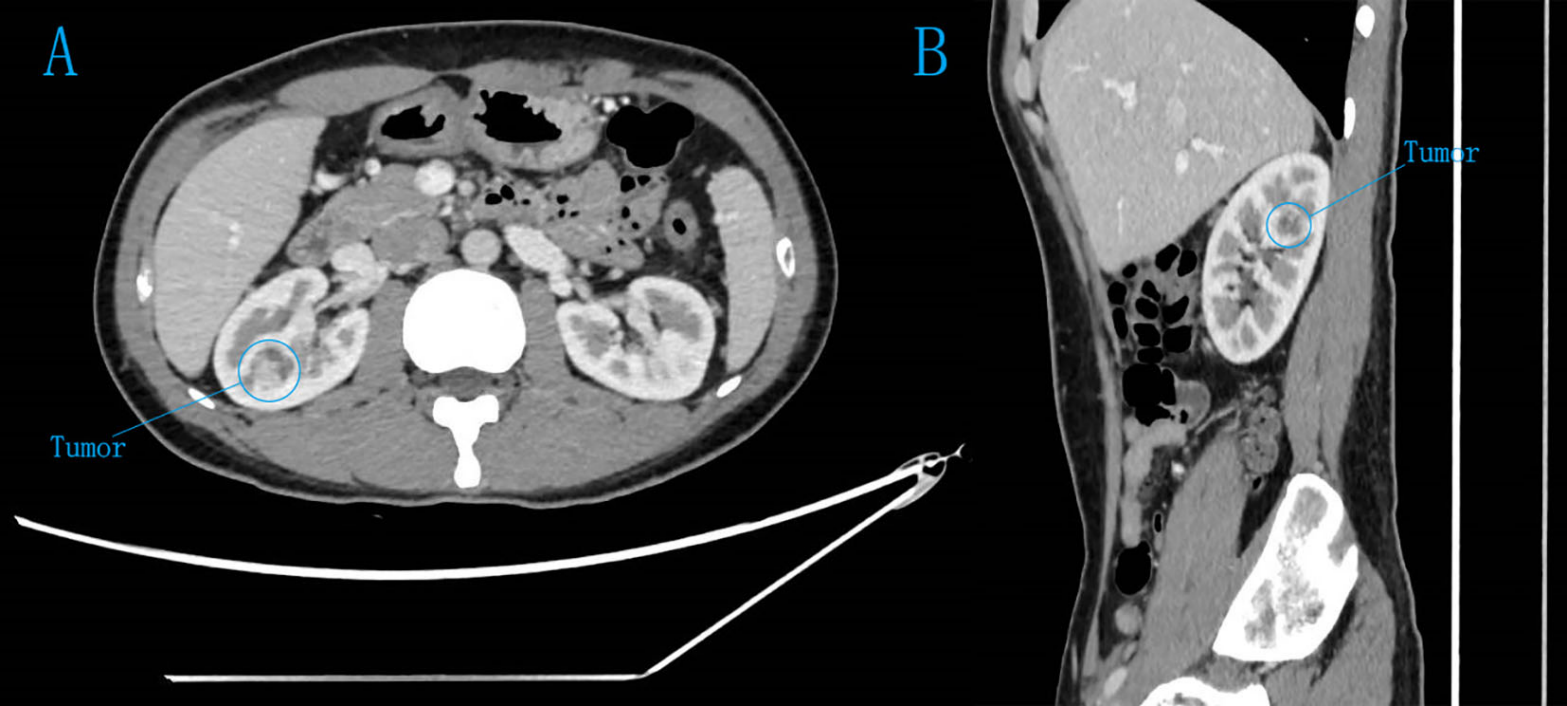
CT plain scan and enhanced images of completely endogenous renal tumours. **(A)** axial view. **(B)** Sagittal view. Contrast-enhanced CT scan of both kidneys in the cortical phase reveals a markedly heterogeneous enhancing mass in the right kidney.

Routine preoperative contrast-enhanced CT or MRI scans were performed. Imaging characteristics showed tumours with a typical “fast-in and fast-out” enhancement pattern (**Figure 1**). Three-dimensional reconstruction confirmed that the tumours were entirely located within the renal parenchyma. Additional preoperative examinations included blood biochemistry, urinalysis, complete blood count, chest X-ray, electrocardiogram, cardiopulmonary function tests, and eGFR assessment.

## 3 Surgical technique

### 3.1 Holographic imaging workflow

Preoperative contrast-enhanced CT/MRI scans were performed using standardized protocols (slice thickness ≤1 mm, intravenous contrast phase). The DICOM data were processed using the Ziwei Dixing Digital Medical holographic reconstruction software, which generated 3D vascular and tumour models with a mean processing time of 50 minutes. During the operation, the surgeons monitored the tumour location and vascular conditions through an external screen, and this step did not increase the total operation time.

### 3.2 Surgical procedure

**Figure 2:**
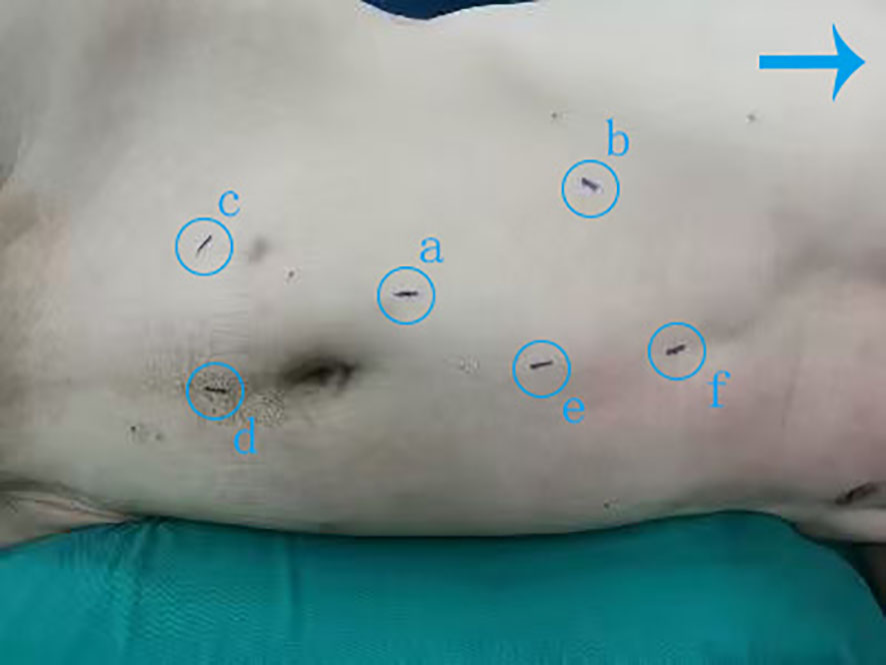
Patient position and cannula puncture site.→ Points toward the patient’s head. **(a)** Camera port(8mm): 1 cm above the upper edge of the umbilicus. **(b)** Robotic arm port 1(8mm): 3 cm below the costal margin on the right midclavicular line. **(c)** Robotic arm port 2(8mm): McBurney’s point. **(d)** Assistant port 1(12mm): 3 cm below the umbilicus. **(e)** Assistant port 2(12mm): 5 cm above the umbilicus. **(f)** Liver retraction port(5mm): 2 cm below the xiphoid process.

**Figure 3:**
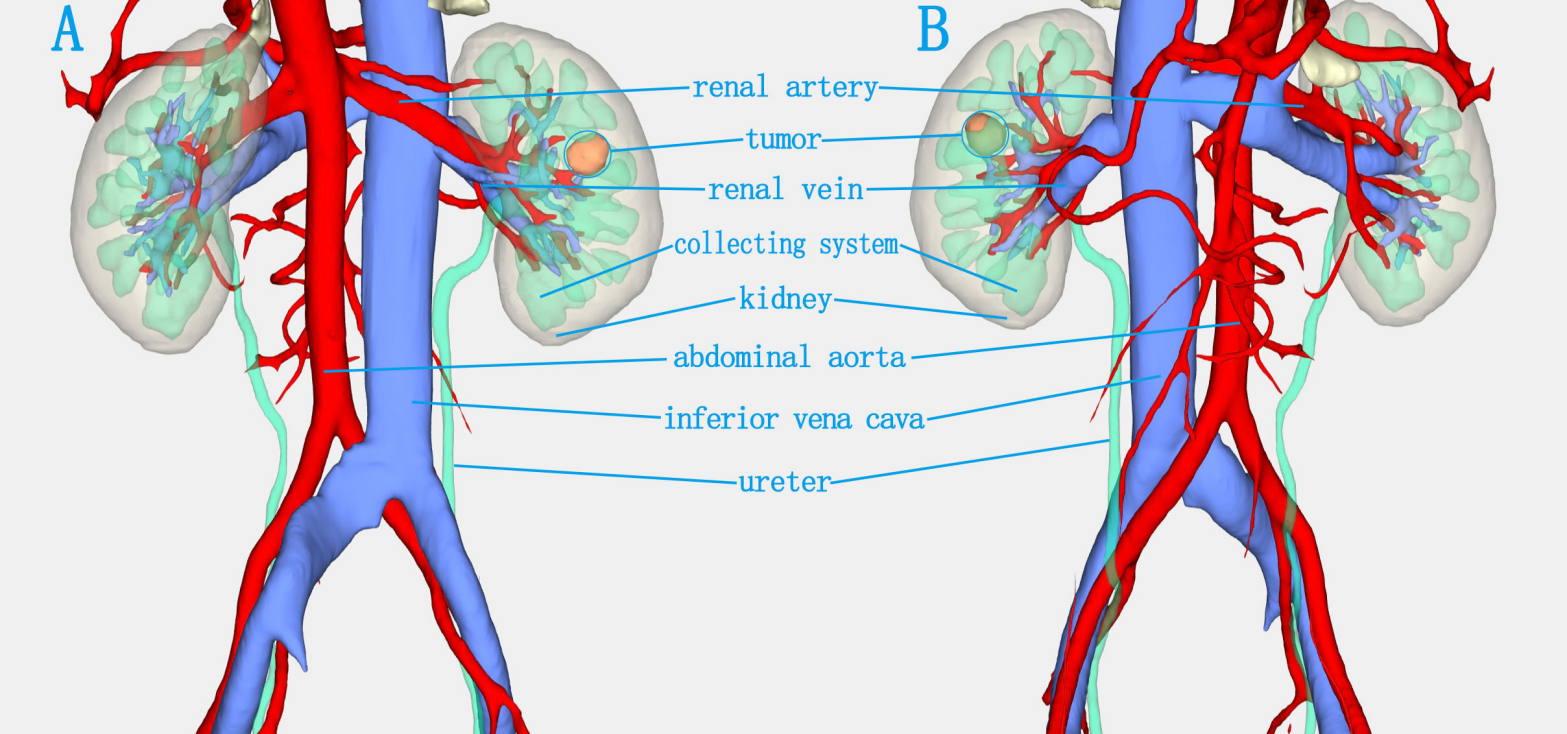
The holographic image of a completely endogenous renal tumour. **(A)** Dorsal view. **(B)** Ventral view. The hologram clearly visualizes the tumor’s position, its spatial relation to adjacent structures, and allows fine dissection of renal vascular branches.

**Figure 4:**
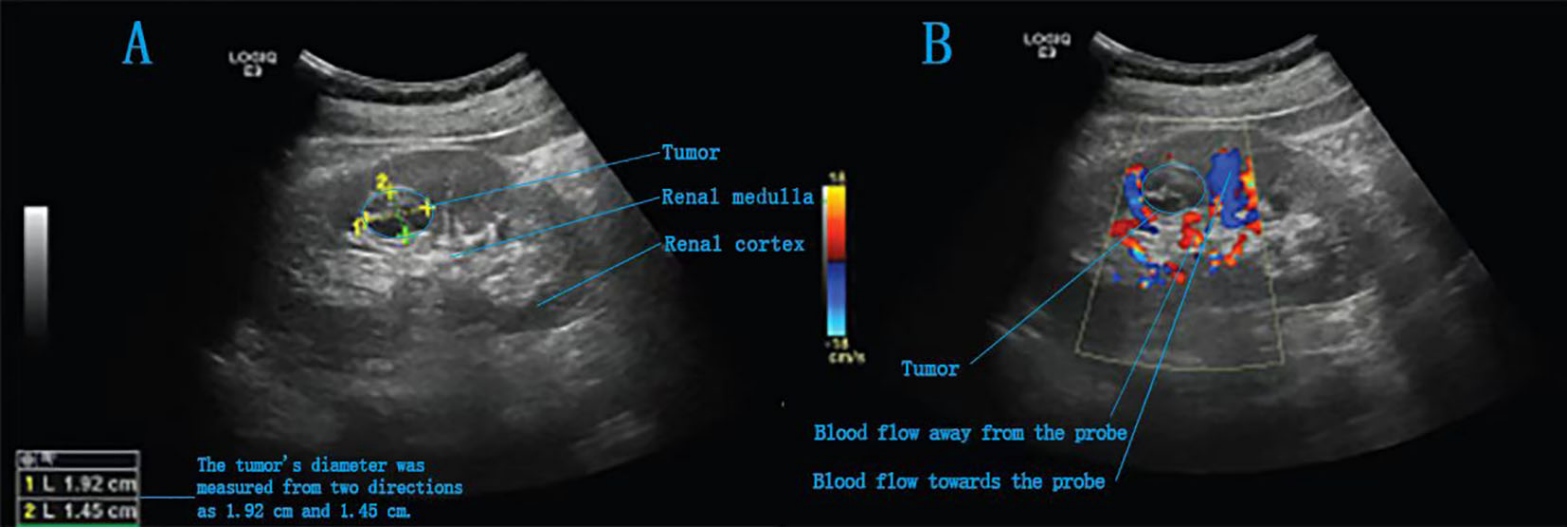
Intraoperative ultrasound images of a completely endogenous renal tumour. **(A)** A slightly hyperechoic area is detected in the right kidney, with heterogeneous internal echotexture. **(B)** Intraoperative Doppler ultrasound image.

The patient was placed in a 70° lateral decubitus position with the lumbar region elevated. One 5-mm, three 8-mm and two 12-mm trocars were inserted into the abdomen for anchoring the Da Vinci robotic system and assisting the surgical team (**Figure 2**). For tumour localization, holographic imaging was first used to identify renal artery branches (**Figure 3**), followed by intraoperative ultrasound confirmation. The ultrasound probe was introduced through a trocar to precisely locate the tumour and delineate its boundaries (**Figure 4**). Monopolar cautery was used to mark the resection margin on the renal surface. The renal artery was clamped with vascular clips, and the tumour was excised along a line 5 mm outside the ultrasound-marked boundary. For renal reconstruction, the first layer was closed using a 3–0 V-Loc continuous suture for the medulla, and the second layer was closed with a 2–0 V-Loc continuous or locked suture for the capsule. Finally, the tumour specimen was retrieved using forceps, and the surgical site was inspected for active bleeding before removing the trocars and placing a drain. The incision was then closed.

### 3.3 Intraoperative blood loss measurement

Intraoperative blood loss was quantified using a combined method: ①Suction canister volume: Total aspirated fluid was collected, and irrigation volume (normal saline used during surgery) was subtracted to calculate net blood loss; ②Gauze weight measurement: Blood-soaked gauzes were weighed (1 g ≈ 1 mL blood), with dry gauze weight as baseline;③Visual estimation: For small residual blood in the surgical field, the surgeon and anaesthesiologist jointly estimated the volume (recorded to the nearest 10 mL).The final blood loss value represented the sum of these three measurements. This protocol was standardized across all cases to ensure consistency.

## 4 Statistical methods

Data analysis was performed using SPSS software. The normality of continuous variables was assessed using the Shapiro-Wilk test (P > 0.10) and Q-Q plots. Based on the distribution type, parametric or non-parametric tests were selected. Normally distributed continuous variables were described as mean ± standard deviation $\bar{x}\pm s$ and compared using t-tests. Non-normally distributed continuous variables were described as median (P25, P75) and compared using the Mann-Whitney U test. Categorical variables were described as frequencies or percentages and compared using chi-square or Fisher’s exact tests (the latter when expected frequencies were <5). SMD were calculated to evaluate the effectiveness of the propensity score matching, with SMD < 0.1 indicating good balance. A P-value < 0.05 was considered statistically significant.

## 5 Outcome measures

The study evaluated perioperative and follow-up data. Intraoperative outcomes included The study evaluated perioperative and medium-term outcomes with a median follow-up of 6 months (range 3-12). Intraoperative outcomes included operative time, estimated blood loss (measured by suction canister volume minus irrigation fluid and weighted gauze), WIT, and conversion to radical nephrectomy. Postoperative outcomes comprised immediate (day 1) serum creatinine levels, pathological staging, surgical margin status (assessed by two blinded pathologists), length of hospital stay, and complications graded by Clavien-Dindo classification (6). Standardized follow-up assessments at 1 and 3 months included serum creatinine, complete blood count, urinalysis, and renal CT to monitor complications (e.g., haemorrhage, urinary fistula), with additional imaging performed as clinically indicated during the 6-month follow-up period.

## 6 Results

The intraoperative and postoperative outcomes of both groups are detailed in **Table 2**.

**Table 2:** Comparison of perioperative and pathological outcomes between RAPN and LPN groups.

Indicator	RAPN group (n=31)	LPN group (n=30)	Statistical value	P-value
Conversion to Radical Nephrectomy	1 (3.2%)	1 (3.3%)	Fisher’s Exact Test	1.000
Warm Ischemia Time (min)	20 [20–25]	25 [20–30]	Z = -2.09	0.037
Intraoperative Blood Loss (ml)	50 [20–50]	50 [50–50]	Z = -2.20	0.028
Operative Time (min)	145 [126–193]	133 [115–163]	Z = -1.63	0.103
Postoperative Hospital Stay (days)	7 [6–7]	7 [5–7]	Z = -1.62	0.105
Complication Rate	3 (9.7%)	2 (6.7%)	Fisher’s Exact Test	1.000
Postoperative Day 1 eGFR (ml/min)	84.7 ± 22.3	87.3 ± 19.9	t = 0.48	0.634
Postoperative 3-Month eGFR (ml/min)	92.2 ± 30.4	95.2 ± 22.9	t = 0.34	0.738
Positive Surgical Margin Rate	0 (0%)	0 (0%)	—	—
Recurrence Rate	0 (0%)	0 (0%)	—	—
Pathological findings				
- Malignant Tumours, n (%)	30 (96.8%)	26 (86.7%)		0.194
Clear cell RCC	27	21		
Papillary RCC	1	2		
Chromophobe RCC	2	3		
- Benign Tumours, n (%)	1 (3.2%)	4 (13.3%)		
Angiomyolipoma	1	1		
Oncocytoma	0	2		
Cystic lesion	0	1		
- WHO/ISUP Grade (for malignant tumours)	(n=29)*	(n=23)*		
Grade 1	4 (13.8%)	0 (0%)		
Grade 2	24 (82.8%)	23 (100%)		
Grade 3	1 (3.4%)	0 (0%)		

**Figure 5:**
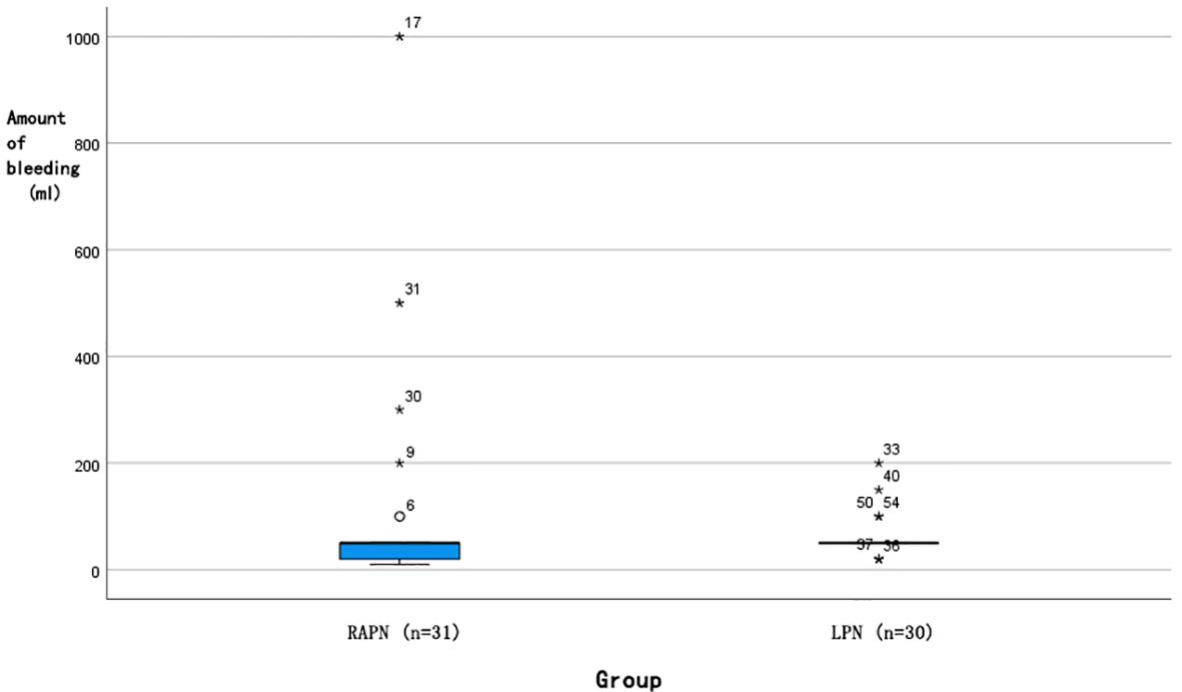
Box plot of intraoperative bleeding distribution for RAPN and LPN surgeries. (█: Median 50 ml; ◯: Outlier; *: *P*<0.05).

Each group had one case converted to radical nephrectomy due to significant intraoperative bleeding. No statistically significant differences were observed in operative time [145 (126, 193) min *vs*. 133 (115, 163) min, *P=*0.103] or postoperative hospital stay [7 (6, 7) days *vs*. 7 (5, 7) days, *P=*0.105]. Analysis of intraoperative blood loss revealed a more favourable distribution in the RAPN group (**Figure 5**), with a median of 50 [IQR 20–50] mL, where 25% of cases had ≤20 mL, compared to the LPN group’s median of 50 [IQR 50–50] mL, with a minimum of 50 mL (Mann-Whitney U test, *P=*0.028). The RAPN group also demonstrated significantly shorter warm ischemia time [20 (20, 25) min *vs*. 25 (20, 30) min, *P=*0.037], with an absolute difference of 5 min (95% CI: 1.2–8.8). In the RAPN group, three perioperative complications occurred, all managed successfully: one Grade IIIa (renal vascular rupture treated with arterial embolization), one Grade II (suspected internal haemorrhage with platelet count of 30×10^9/L and haemoglobin of 66 g/L, treated with transfusion), and one Grade II (chylous leakage managed with nutritional support). The LPN group had two complications: one Grade V (acute myocardial infarction with fatal outcome) and one Grade II (suspected pulmonary embolism treated with anticoagulation). Complication rates did not differ significantly between groups (*P>*0.05). All malignant tumours were further classified according to the World Health Organization/International Society of Urological Pathology (WHO/ISUP) grading system. In the RAPN group, among the 30 malignant cases, the distribution of WHO/ISUP grades was: Grade 1 (n=4, 13.3%), Grade 2 (n=24, 80.0%), and Grade 3 (n=1, 3.3%); one case of chromophobe renal cell carcinoma (RCC) was not graded by this system. In the LPN group, all 26 malignant cases were classified as Grade 2 (100%); two cases of chromophobe RCC and one case of papillary RCC were not graded. The proportion of benign tumours was comparable between groups (3.2% *vs*. 13.3%, P = 0.194). All cases achieved negative surgical margins. No significant differences were noted in postoperative eGFR at day 1 [(84.67 ± 22.25) mL/min *vs*. (87.26 ± 19.92) mL/min, *P=*0.634] or 3 months [(92.17 ± 30.42) mL/min *vs*. (95.21 ± 22.91) mL/min, *P=*0.738]. At a median follow-up of 6 months (range 3-12), no local recurrence or distant metastasis was detected on imaging in either group.

## 7 Discussion

Following rigorous propensity score matching, the RAPN and LPN groups in our study demonstrated excellent balance in all key baseline and tumour characteristics. This enhances the validity of our subsequent findings, as the observed differences in perioperative outcomes are less likely to be attributable to pre-existing patient or tumour factors.

The present study demonstrates that holographic imaging combined with real-time ultrasound-guided RAPN significantly reduces WIT and intraoperative blood loss compared to conventional LPN in the treatment of completely endophytic renal tumours, while maintaining comparable operative times, complication rates, and renal functional outcomes (4, 5). These findings highlight the potential advantages of integrating advanced imaging technologies with robotic assistance for complex renal surgery, particularly in cases where visual cues are minimal due to the complete parenchymal encapsulation of the tumour.

Our results showed a statistically significant reduction in WIT in the RAPN group (20 min *vs*. 25 min, P = 0.037), representing a clinically relevant 20% reduction in ischemia time. This finding is consistent with prior studies investigating robotic versus laparoscopic approaches for complex renal masses. Gu et al. (4) reported a mean WIT of 22.5 minutes in RAPN versus 28.2 minutes in LPN for completely endophytic tumours, supporting the notion that robotic assistance facilitates quicker vascular control and resection. The articulated instruments and three-dimensional visualization of the Da Vinci system may allow more precise dissection and suturing, thereby reducing ischemic intervals. Similarly, Chung et al. (5) emphasized the technical challenges of LPN in completely intraparenchymal tumours, often resulting in prolonged WIT due to difficulties in tumour localization and resection plane identification. Our findings align with these reports and further suggest that the integration of holographic imaging may enhance preoperative planning and intraoperative navigation, contributing to more efficient clamp management and reduced ischemic burden.

Notably, intraoperative blood loss was significantly lower in the RAPN group, with 25% of cases achieving blood loss ≤20 mL—an outcome not observed in the LPN group where the minimum blood loss was 50 mL. This advantage may be attributed to the improved dexterity and stability offered by the robotic system, enabling finer dissection and more controlled haemostasis during tumour enucleation. The tremor filtration and motion scaling capabilities of the robotic platform likely facilitate precise dissection along the tumour-parenchyma interface, minimizing vascular injury. Froghi et al. (6) also noted a trend toward reduced blood loss in RAPN compared to LPN for small renal masses, although the difference did not always reach significance across all studies. Our cohort, strictly limited to completely endophytic tumours (R.E.N.A.L. score E = 3), may represent a more homogenous and technically challenging population, thereby magnifying the benefits of robotic precision in vascular control.

Interestingly, we found no significant difference in operative time between the two groups (145 min for RAPN *vs*. 133 min for LPN, P = 0.103). This is consistent with Zhang et al. (4), who reported comparable operative times between RAPN and LPN for complex renal tumours. The additional time required for docking the robotic system and setting up the holographic imaging interface may have been offset by the efficiency gained during tumour excision and renorrhaphy phases of the procedure. Moreover, the use of holographic imaging and intraoperative ultrasound in both groups likely contributed to accurate tumour localization, minimizing unnecessary dissection and potentially equalizing the operative duration between approaches.

Regarding renal functional outcomes, no significant differences were observed in eGFR at postoperative day 1 or at 3 months. This suggests that both techniques, when supported by advanced imaging guidance, can effectively preserve renal function despite the technical challenges posed by completely endophytic tumours. These results are in line with those of Michiels et al. (7), who reported no significant difference in postoperative renal function between image-guided RAPN and conventional approaches. The combination of reduced WIT and precise excision under imaging guidance may help mitigate ischemic injury and parenchymal loss, thereby protecting renal function even in complex cases (8, 9). The comparable functional outcomes also suggest that the potential benefits of robotic precision in tissue preservation may be balanced by the learning curve associated with this technology.

Both groups achieved 100% negative surgical margins and no recurrence during follow-up, underscoring the oncologic safety of both techniques when performed by experienced surgeons with appropriate imaging guidance. Notably, this oncologic efficacy was achieved despite a comparable yet slightly more diverse pathological profile in the RAPN group, which included cases of both Grade 1 and Grade 3 disease. These outcomes are comparable to those reported in previous studies (4–7) and highlight the critical role of intraoperative imaging in achieving complete tumour resection (10). The combination of holographic imaging for preoperative planning and real-time ultrasound for intraoperative confirmation appears to provide complementary benefits for ensuring oncologic adequacy regardless of surgical approach.

This study has several important limitations that warrant consideration. First, and most critically from a methodological standpoint, the integrated application of holographic imaging across both the RAPN and LPN groups, while ensuring a fair comparison of the surgical platforms under identical navigational conditions, prevents a definitive disaggregation of the benefits uniquely attributable to the robotic system from those conferred by holographic imaging itself. A skeptical reader could rightly argue that the observed advantages stem primarily from the Da Vinci platform, with holography playing a secondary role. Although we deliberately employed this “all-in” imaging design to reflect real-world clinical practice for complex cases and to test the synergistic value of the combined approach, we acknowledge that a multi-arm randomized controlled trial including a RAPN cohort without holographic guidance would be necessary to isolate the independent contributions of each technology. Despite this constraint, our findings robustly demonstrate the superior performance of the robotic platform when augmented with advanced imaging for this specific tumour type. Second, while our sample size (n=61) provided adequate power for primary outcomes like WIT, it was underpowered for rare secondary endpoints (e.g., complication rates) due to the rarity of completely endophytic tumours (R.E.N.A.L score E = 3). Third, with a median follow-up of 6 months (range 3-12), our study is insufficient to evaluate long-term oncologic outcomes such as recurrence or metastasis (typically occurring beyond 2 years in renal cell carcinoma), nor does it capture delayed complications (e.g., chronic kidney disease). Fourth, all procedures were performed by an experienced robotic surgery team, which while ensuring technical consistency may not reflect outcomes during the learning curve phase at other institutions. Fifth, we lacked formal cost-effectiveness analysis comparing RAPN versus LPN, particularly regarding the additional expenses of holographic imaging and robotic platforms. Finally, while emerging technologies like ICG fluorescence imaging (11), augmented reality (12), and 3D printing (13) show promise, their comparative value requires further study. A multicentre randomized trial with extended follow-up (≥3 years) and economic evaluation is needed to validate these findings across diverse clinical settings.

## 8 Conclusion

The surgical management of completely endophytic renal tumours presents significant technical challenges in urologic practice. Our findings demonstrate that holographic imaging combined with real-time ultrasound guidance in RAPN, when performed by experienced surgeons, offers superior perioperative outcomes - particularly in reducing warm ischemia time and intraoperative blood loss - compared to conventional LPN. This integrated imaging approach enhances surgical planning by enabling precise tumour localization and vascular mapping, thereby potentially minimizing complication risks. While these technological advancements in imaging and robotic surgery show promise for optimizing patient outcomes, further validation through large-scale, prospective randomized controlled trials with long-term follow-up is warranted to confirm these preliminary results.

## Data Availability

The original contributions presented in the study are included in the article/**Supplementary Material**. Further inquiries can be directed to the corresponding author.

## Supplementary material

Supplementary Figure 1 CONSORT-style flowchart illustrating patient screening, exclusion, and enrollment.

Supplementary Table 1 Characteristics and Exclusion Reasons for the Two Unmatched Patients. (Note on R.E.N.A.L. Score: The suffix ‘x’ indicates that the posteriorly located tumour could not be assigned an ‘a’ (anterior) or ‘p’ (posterior) designation.).

Supplementary Table 2 Comparison of Baseline Characteristics Between Excluded Patients and the Final Included Cohort. (P-values were calculated using independent t-test, Mann-Whitney U test, or Fisher’s exact test, as appropriate. No statistically significant differences were observed (all P > 0.05), indicating minimal selection bias from the exclusion of these patients.).
