# Correction: Application of holographic imaging combined with real-time ultrasound-guided robot-assisted partial nephrectomy in the treatment of completely endophytic renal tumours: a retrospective cohort study comparing with pure laparoscopic surgery

**DOI:** 10.3389/fonc.2025.1755648

**Published:** 2025-12-18

**Authors:** Tongbin Gao, Yuan Gao, Dawei Wang, Yongchun Yu, Zhentao Zhang, Feihu Tang

**Affiliations:** 1 Weifang People’s Hospital, Urology Medical Center, Weifang, China; 2 Affiliated Hospital of Shandong Second Medical University, School of Clinical Medicine, Shandong Second Medical University, Weifang, China

**Keywords:** completely endophytic renal tumour, da Vinci robotic system, holographic imaging technology, intraoperative ultrasound, minimally invasive surgery

The order of authors in the author list of the published paper was erroneously given as:

“Feihu Tang^1^*, Tongbin Gao^2^, Yuan Gao^2^, Dawei Wang^2^, Yongchun Yu^2^ and Zhentao Zhang^1^

1 Affiliated Hospital of Shandong Second Medical University, School of Clinical Medicine, Shandong Second Medical University, Weifang, China

2 Weifang People’s Hospital, Urology Medical Center, Weifang, China”

The correct author list reads:

“Tongbin Gao^1^, Yuan Gao^1^, Dawei Wang^1^, Yongchun Yu^1^, Zhentao Zhang^2^ and Feihu Tang^2^*

1 Weifang People’s Hospital, Urology Medical Center, Weifang, China

2 Affiliated Hospital of Shandong Second Medical University, School of Clinical Medicine, Shandong Second Medical University, Weifang, China”

The original version of this article has been updated.

