# Supplementary figures and images for: Application of holographic imaging combined with real-time ultrasound-guided robot-assisted partial nephrectomy in the treatment of completely endophytic renal tumours: a retrospective cohort study comparing with pure laparoscopic surgery
Source: Front Oncol. 2025 Nov 24;15:1671896. doi: 10.3389/fonc.2025.1671896 (PMC12682635; doi:10.3389/fonc.2025.1671896)

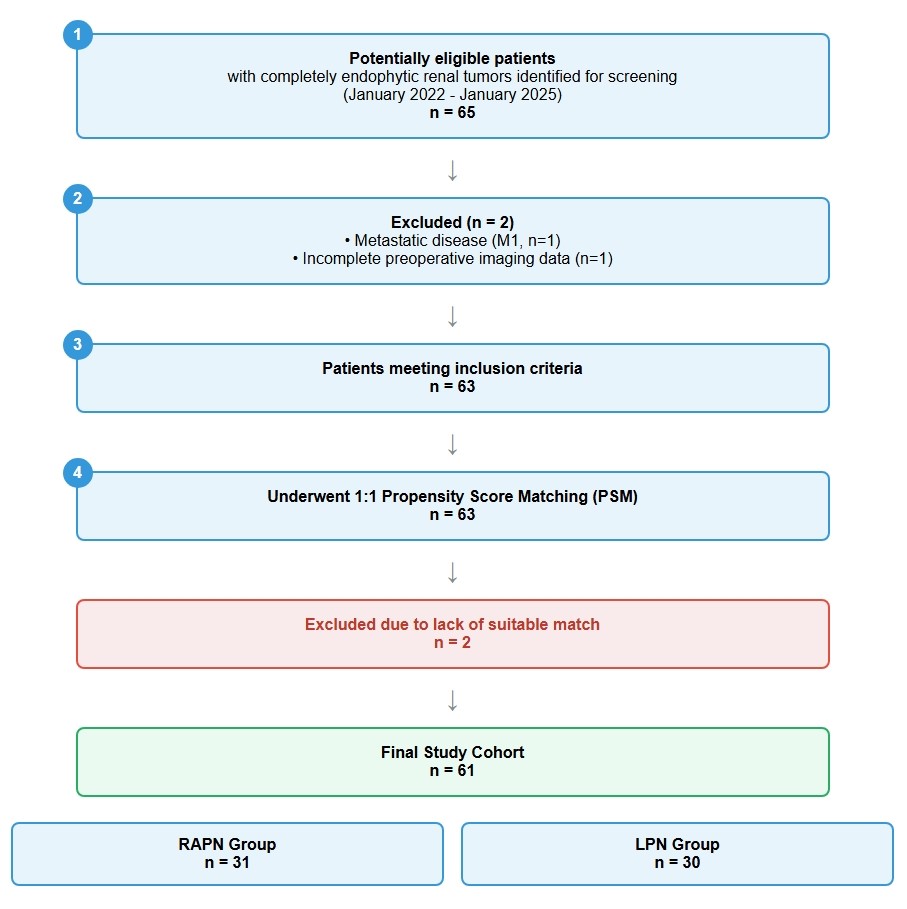

Supplement: Supplementary Figure 1 — CONSORT-style flowchart illustrating patient screening, exclusion, and enrollment. [file Image1.jpeg]
